# Study protocol for a controlled trial of a resilience program on psychological distress in correctional officers in Australia

**DOI:** 10.1186/s12888-023-04592-4

**Published:** 2023-02-09

**Authors:** Dharani Keyan, Katie S. Dawson, Richard A. Bryant

**Affiliations:** School of Psychology, University of New South Wales, Sydney, Australia

**Keywords:** Resilience, Prevention, Workplace mental health

## Abstract

**Background:**

The mental health impacts of workers within correctional settings has been of increasing focus over the past number of years. This paper outlines the study protocol for a trial that tests the efficacy of a brief resilience program, relative to a no intervention control in reducing general psychological distress and absenteeism in a cohort of correctional personnel in NSW, Australia.

**Methods:**

A, parallel, randomized controlled trial will be carried out in a small group format. Following informed consent, corrective personnel within prisons across NSW will volunteer to either attend a clinician delivered resilience program on stress management skills or not (*N* = 600). The primary outcome will be change in psychological distress including anxiety and depression at 2-months post intervention. Secondary outcomes include help-seeking behaviours and absenteeism.

**Discussion:**

This prevention focused treatment trial will assess whether a brief clinician delivered resilience program will reduce absenteeism and mitigate psychological distress in a cohort of corrective personnel within NSW, Australia. This study will yield insights into the role of a brief psychological program in mitigating the psychological distress reported by personnel in correctional settings.

**Trial registration:**

This trial was prospectively registered on the Australian New Zealand Clinical Trials Registry (ACTRN12622000029796).

**Ethics and dissemination:**

Ethics approval has been obtained from University of New South Wales Human Research Ethics Committee. Results of the trial will be submitted for publication in peer reviewed journals and findings presented at scientific conferences and to key service providers and policy makers.

## Background

Repeated exposure to threatening environments involving critical incidents and potentially traumatic events take a toll on one’s mental health. Research on the wellbeing of public safety personnel such as police, emergency service and correctional workers has been an increasing focus in recent years [1]. The nature of attending to critical events including death, threatened and actual violence, verbal altercations, self-injury, and inmate overcrowding on a routine basis can adversely impact mental health. One Canadian study revealed that up to one quarter of police, emergency service and corrections personnel screened positive for symptoms for post-trauma stress disorder and major depression [2]. These estimates parallel other international estimates of displaying that up one third of these personnel display mental health problems that meet diagnostic criteria [3, 4]. In particular, an increased frequency and duration of exposure to threatening environments can worsen health and mental health outcomes [5]. To this end, higher perceptions of threat and unpredictability reported by corrections personnel (than personnel in counterpart public safety occupations) may also contribute to this relationship between exposure to potentially traumatic events and negative mental health incidents [6]. Apart from heightened rates of mental disorders in corrective personnel [2], this cohort also reports suffering from physical ill health (i.e., stress related illness, ulcers, increased blood pressure), heightened work-home conflict (e.g., difficulties in separating work from home, fatigue, reduced time for family), job dissatisfaction and burnout [7, 8]. In turn, considerable monetary costs are associated with compensation claims owing to serious mental health conditions and related lost productivity and short and long-term absenteeism [9, 10].

Furthermore, challenges with the correctional organisation that are often cited provide a keen insight into the psychosocial environments surrounding corrective personnel. To this end, qualitative studies have highlighted that the nature of insecure employment (i.e., casual work), shift work, dissatisfaction with wages and interpersonal challenges with management [3, 11] can together add to sources of stress in corrective personnel. Here, reduced access to quality mental health care within the organizational structure, stigma, and perceptions of breach of confidentiality can together act as strong barriers to help-seeking [12]. To this end, the development of mental health interventions for this cohort must address help-seeking barriers and endeavour to work alongside the organizational and structural challenges such that meaningful change in mental health outcomes can be sought. Supporting this proposition is qualitative research suggesting that organizational acknowledgment of mental health challenges resulting from adversity within the workplace, coupled with the provision of mental health awareness and first aid training and programs for workers, are in keen demand as reported by workers [3]. As such, mental health programs that are supported by organizational structures may elicit engagement and motivation from corrective personnel, and in turn, provide a path to addressing important mental health challenges of this population.

The current study will assess the efficacy of a novel resilience training program that builds on a program developed by the World Health Organization (WHO) [13] to teach awareness about identifying stress, stress reduction strategies, problem management, and seeking/maintaining social support and connection. The program will be conducted in collaboration with Corrective Services New South Wales (CSNSW) and will be implemented within prison settings, wherein participation will be voluntary. This program will be compared against a no intervention control group that comprises repeated assessments to assess the efficacy of the resilience group overtime. This form of control condition was adopted because there is typically no resilience or stress reduction programs available in CSNSW for custodial staff, and so the rationale for this comparator was to test the efficacy of the intervention relative to current practices. In summary, this project takes a prevention perspective to test the efficacy of a potentially scalable program to reduce mental health problems in high-risk occupations, such as correctional facilities.

## Methods

### Aims & hypotheses

This study will assess the efficacy of a novel resilience training program supported with use of an app to practise learned stress reduction strategies. This Resilience Program is an adapted version of WHO’s Problem Management Plus (PM +) program [13], and designed from a prevention perspective to provide mental health awareness, and brief strategies for stress reduction in a cohort of corrective personnel across Australia. The randomized controlled trial (RCT) will compare this brief training program against a no intervention control group that will only undertake assessments at critical timepoints. Those receiving this resilience program are hypothesised to report lower levels of psychological distress, reduced absenteeism and increased help seeking relative to the a no intervention arm. The primary outcome measure is the Kessler Psychological Distress Scale-10 item version [14].

### Design & setting

**Fig. 1:**
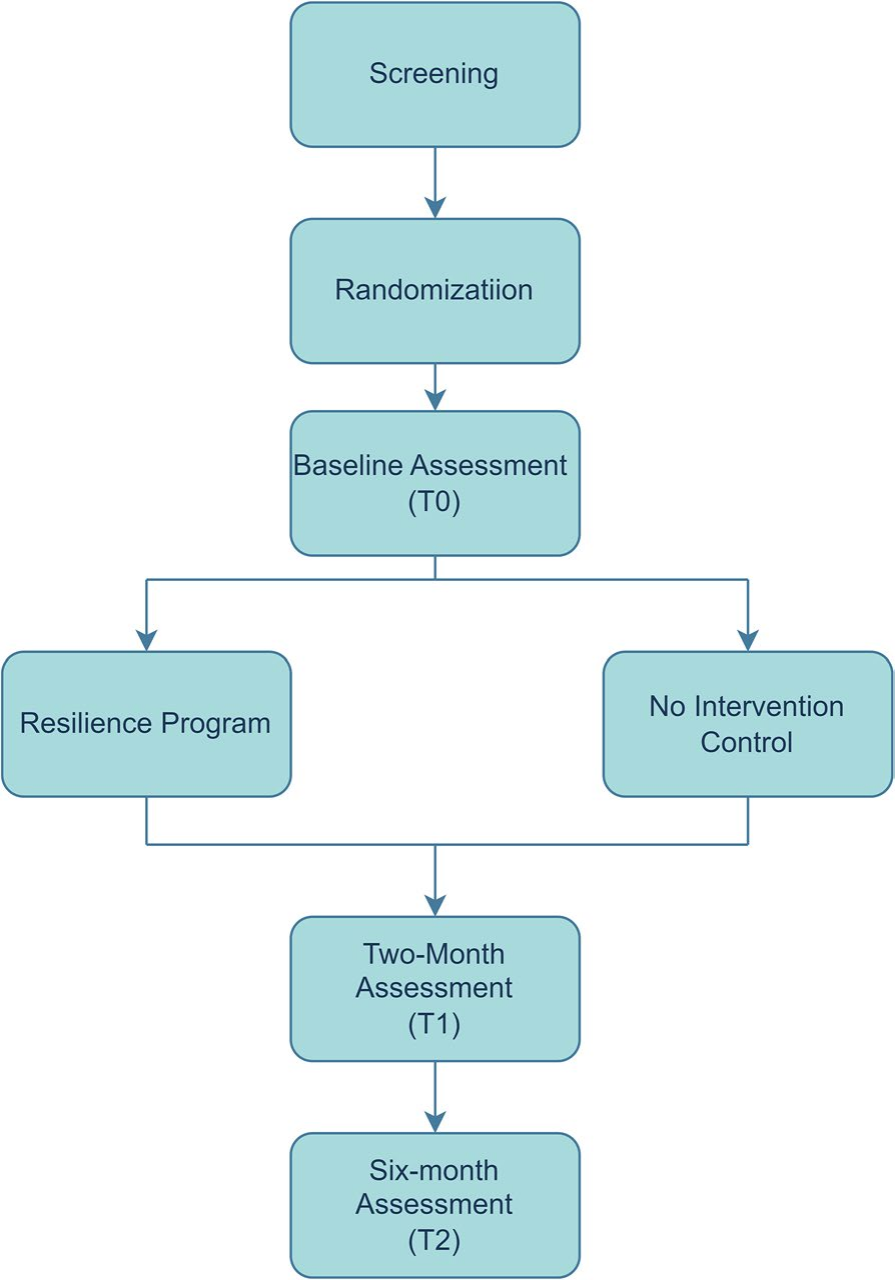
Flow diagram of study

This study is designed as a parallel RCT that will be conducted in prisons across NSW in Australia. The study will deploy a 2 (Treatment condition) × 3 (Assessment Point) factorial design. Participants will be assessed at baseline (T0),, at two-month follow-up (T1), and at six-month follow-up (T2), where T1 will be the primary outcome timepoint (see Fig. 1). This trial was registered on Australian New Zealand Clinical Trials Registry (ACTRN12622000029796) on 14 January 2022 and received ethical approval from the University of New South Wales Human Ethics Panel C, Australia. The Resilience Program will provide 2 × 2-h sessions administered by clinical psychologists one month apart, delivered in groups of 10–15 people.

### Participants, recruitment and informed consent

Six hundred Corrective Services personnel will be identified through prisons across NSW. Participants will be of minimum 18 years of age and currently be employed by Correctional Services NSW. As this is a preventative intervention, the presence of psychological distress will not be a requirement of entry into this trial. Informed consent will be obtained both verbally and in written form, where the former will be essential prior to personnel taking part in the resilience training session. At the time of written consent, participants will complete the pre-assessment (T0) consisting of the K10 and help-seeking behaviour. Recruitment commenced in March 2022.

### Randomization

Prison personnel within eight prison settings across NSW will be allocated to either the resilience program or no control intervention on a 1:1 basis (no block randomization). This will be carried out on a computerized software by a research assistant who is independent to any other aspects of the intervention. This randomization will take place prior to the completion of the T0 assessment. No blinding will be used for the participants and clinical psychologists delivering the interventions.

### Intervention

#### Resilience program

The Resilience program will be administered in a face-to-face group format consisting of 10–15 people over two 2-h group sessions (one month apart). These sessions will consist of an educational and group discussion format with content consisting of stress coping strategies delivered by clinical psychologists: anxiety reduction, problem solving, behavioral activation, and accessing social support. Following the sessions, participants will be instructed to download an app, named *Headgear* [15] that was developed by the BlackDog Institute to support resilience building and better wellbeing. This app will provide additional skills in anxiety reduction, mindfulness, and behavioural activation. This app will function as an ongoing self-help aid to be used at the participants’ discretion. Specifically, instructional prompts in this app will guide participants’ use of strategies and this will be available in an ongoing basis for the duration of the study. Adherence will be assessed by monitoring participants' attendance at each session via a checklist maintained by the clinical psychologist.

#### No intervention

The control condition will engage in repeated assessments across T0, T1, and T2.

### Primary outcome

The Kessler-10 (K-10) is a well validated indicator of psychological distress [14] and comprises of 10 questions about general wellbeing, experience of emotional states including symptoms of depression and anxiety over the past four weeks. Items are scored on a 5-point scale (range 0 to 50; higher scores indicate higher levels psychological distress), where a cut off of 20 [16] will be used to indicate the presence of psychological distress.

### Secondary outcomes

Participants will self-report their help-seeking behaviours using a single dichotomous questions asking if participants have sought assistance for mental health issues in the past six months. The trial will also assess absenteeism as measured by sickness leave from Department of Corrective Service work records, where this will be collected at T2*.*

### Data oversight & management

Study implementation procedures will be monitored on an ongoing basis by the trial management committee consisting of the principal investigator, co-investigators, and local research coordinators. All adverse events that take place during the trial will be monitored by the facilitators at each session, whereby any reported adverse events will be referred to the UNSW Human Ethics Committee and Trial Sponsor. Further, assessments using the K10 were used to detect severe distress and relevant participants were contacted and recommended referral resources.

All participants will be required to have their identities known to the study as they will be followed up for trial assessments at two- and six-month follow-up assessments (T1 & T2). All data from pre-treatment, and follow-up online surveys will be entered on an electronic database in a deidentified manner in which participants are allocated a unique identification number. Restricted access will be made available to research staff directly involved in the study. AES-256 bit encryption will be used move any data from one electronic location to another. The research staff working on the project will initially have access to the data via the UNSW Research Long Term Data Store Interface. Any study protocol amendments will be approved by the local ethics committee prior to implementation. All investigators and participants enrolled in the trial will be informed.

### Analysis

Personnel analysing the data from this trial will be blinded to group allocation. A total of 600 Corrective Services Personnel will be included in the trial. Power calculations were conducted using GPower 3.1.9.7 to determine the between treatment condition effect at follow-up on the premise of a small effect size. This calculation indicated a minimum sample size of 270 participants per group (power = 0.95, alpha = 0.05, two-sided). On the basis of 10% attrition at follow-up, it was estimated that 600 participants (300 per group) were needed.

Analyses will focus primarily on intent-to-treat analysis. Using SPSS version 28, hierarchical linear mixed models will be used to study differential effects of each treatment condition because this method effectively handles missing data by calculating estimates of trajectories. Fixed (intervention, time of assessment) effects and their interactions will be considered in the unstructured models, which will provide an index of the relative effects of the treatments; time of assessment will include baseline, posttreatment, and 3-month follow-up. For the follow-up analyses between the two conditions, analyses will focus on linear time effects, treatment conditions, and interactions. Fixed effects parameters were tested with the Wald test (t-test, *p* < 0.05, two-sided) and 95% confidence intervals. Cohen’s (d) effect size was calculated for all analyses. The primary outcome measure will be the K10. The primary outcome timepoint will be the 6 months assessment. As baseline assessments are incorporated into this modelling, the likelihood of no data is zero. To this end, missingness will be tested at random by assessing baseline characteristics of those do and do not drop out from the study.

### Ethics

This project has been approved by the UNSW Human Research Ethics Committee.

## Discussion

Evaluation of this novel resilience training program in Correctional personnel in the Australian Population will address the significant and growing mental health needs of this population. Specifically, this intervention trial will address the potential for a brief scalable program in reducing psychological distress and absenteeism in this cohort. If found to be effective, this resilience program may be rolled out to other affected public safety personnel settings globally for further adaptation.

## Data Availability

This report is a protocol paper and so no data is involved. The datasets used and/or analysed upon completion of the current study will be available from the corresponding author on reasonable request.

## Abbreviations

AES: Advanced Encryption Standard
HLM: Hierarchical linear mixed models
K-10: Kessler 10
NSW: New South Wales
PM +: Problem Management Plus
RCT: Randomized controlled trial
WHO: World Health Organization
